# Hospital Festivals and Meetings

**Published:** 1894-07-14

**Authors:** 


### HOSPITAL FESTIVALS AND MEETINGS.

### POPLAR HOSPITAL FOR ACCIDENTS.

The thirty-ninth annual festival dinner in aid of the Poplar Hospital for Accidents was held on July 3rd at the Holborn Restaurant, Mr. C. W. Cayzer, M.P., presiding. Lord Knutsford, Sir Thomas Fowler, the Hon. Sydney Holland, Sir Donald Currie, and Colonel Du Plat Taylor were amongst those present. After the usual loyal toasts, Lord Knutsford proposed "The Medical Staff," coupling with the toast the names of Mr. F. M. Corner, Mr. M. Brownfield, and Mr. W. Radford. In proposing the toast of the evening, "Success to the Poplar Hospital," the Chairman reminded his hearers that the old building, situated in a most crowded dock district, had done its work for thirty-eight years, and the new institution was opened three weeks ago by the Prince of Wales. The want of accommodation had previously been most severely felt. The new arrangements enabled them to deal with all classes of accidents. As many as 364 persons had been treated for accidents in a week, while the average number of such cases a year amounted to 14,000. The building had cost upwards of £30,000, and was entirely out of debt. The list of subscriptions would have to be increased to the extent of £1,000 a-year to meet the expenditure, and he expressed confidence that this amount would be secured, and that the institution would continue to do great good in a neighbourhood where it was badly wanted. The Hon. Sydney Holland and Sir Donald Smith replied. Subscriptions amounting to nearly £2,000 were afterwards announced.

### HOMES FOR WORKING BOYS.

A meeting in aid of the funds for these excellent homes was held on Thursday last at the Mansion House. The Duke of Fife presided, and told how the work had been carried on now for twenty-three years—the good work of endeavouring to save struggling lads from the dangers and temptations of life in a great city. He appealed for funds to enable the homes to be successfully maintained; £1,500 was required to clear off an existing debt and continue the work to the end of the year.

Archdeacon Farrar spoke of the value of these homes for boys working in London, saying he took a deep interest in the institution, and believed it to be worthy of all support. The social condition of life in England at the present day, he said, gave rise to feelings of uneasiness. Great cities were the graves of physique and morality, and the utmost must be done for future generations. Prevention was better than cure, and in supporting institutions of this kind they were rendering the highest possible benefit to posterity.

Mr. A. J. Balfour, M.P., dwelt on the reasons why it was incumbent upon those interested in the welfare of London and its future citizens to do something in support of these homes for youths. It was obvious members of a Christian community that to provide a substitute for home for the homeless was a good thing. It was the special duty of those whose business and profit called them day by day into the City to support this institution, because this task was the necessary consequence of the development of the industries of London, and the new social conditions. It was a humane work and one that would bear fruits in the future, while in the present it would relieve suffering and diminish vice. He very earnestly recommended this most excellent charity. Addresses by Dr. Pentecost, Mr. Quintin Hogg, and Mr. Pelham, and a vote of thanks to the chairman concluded the proceedings.

